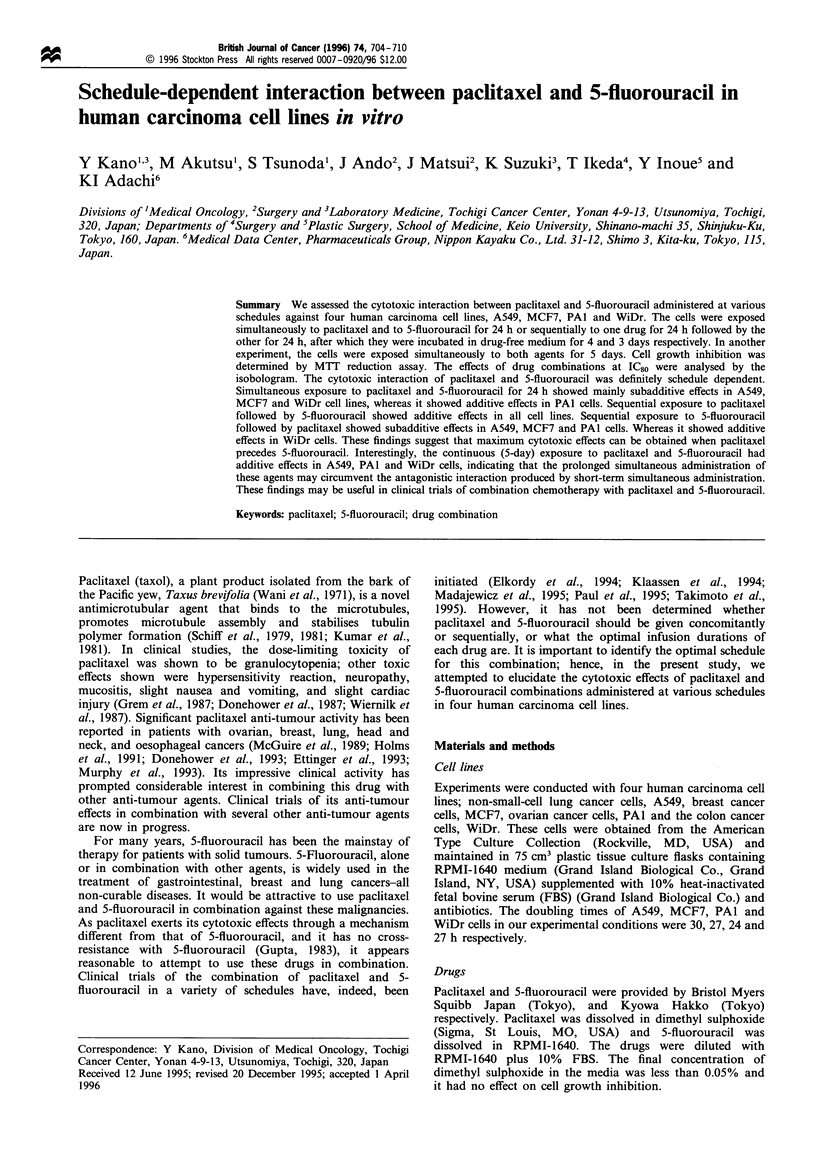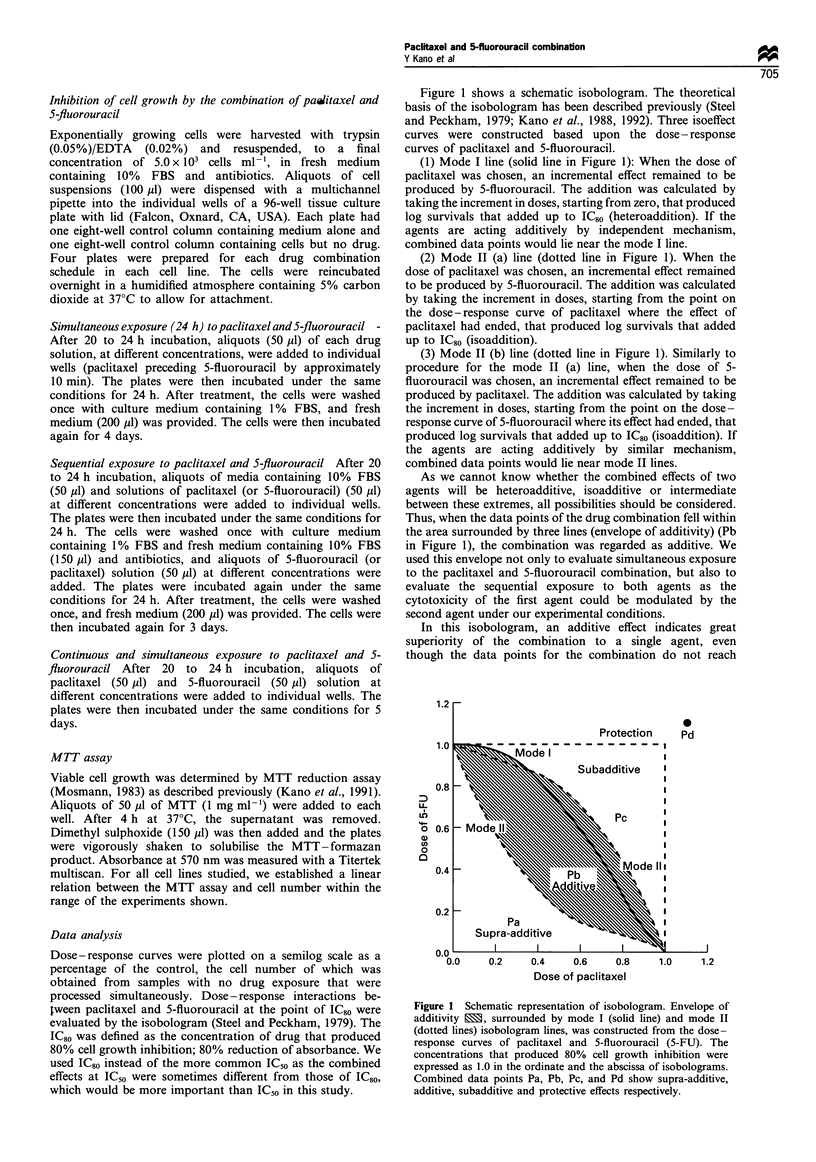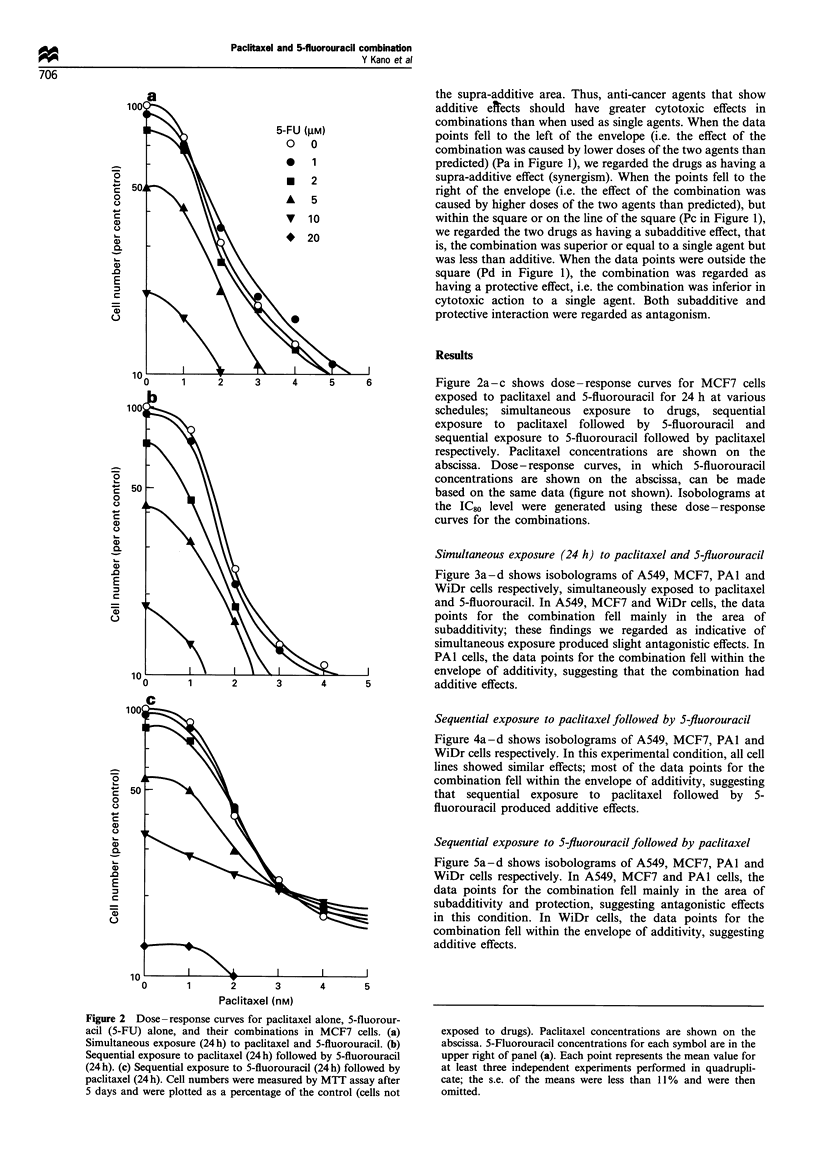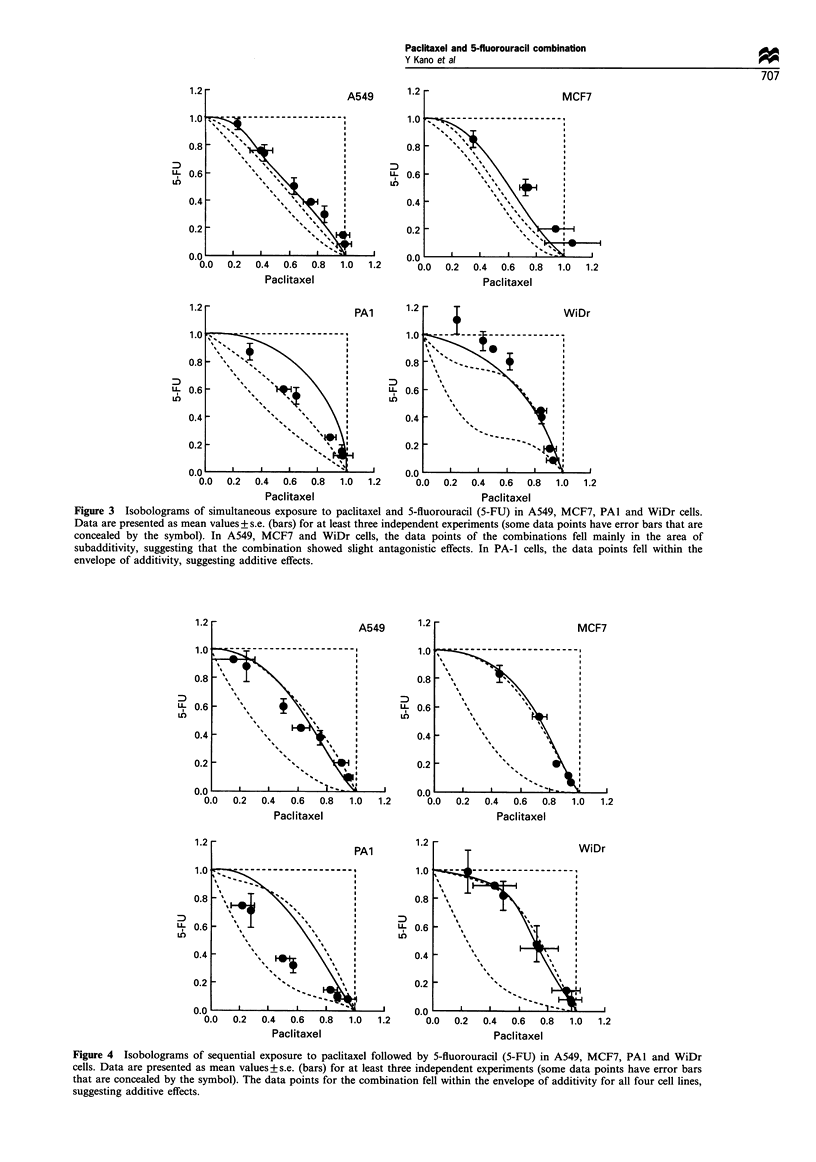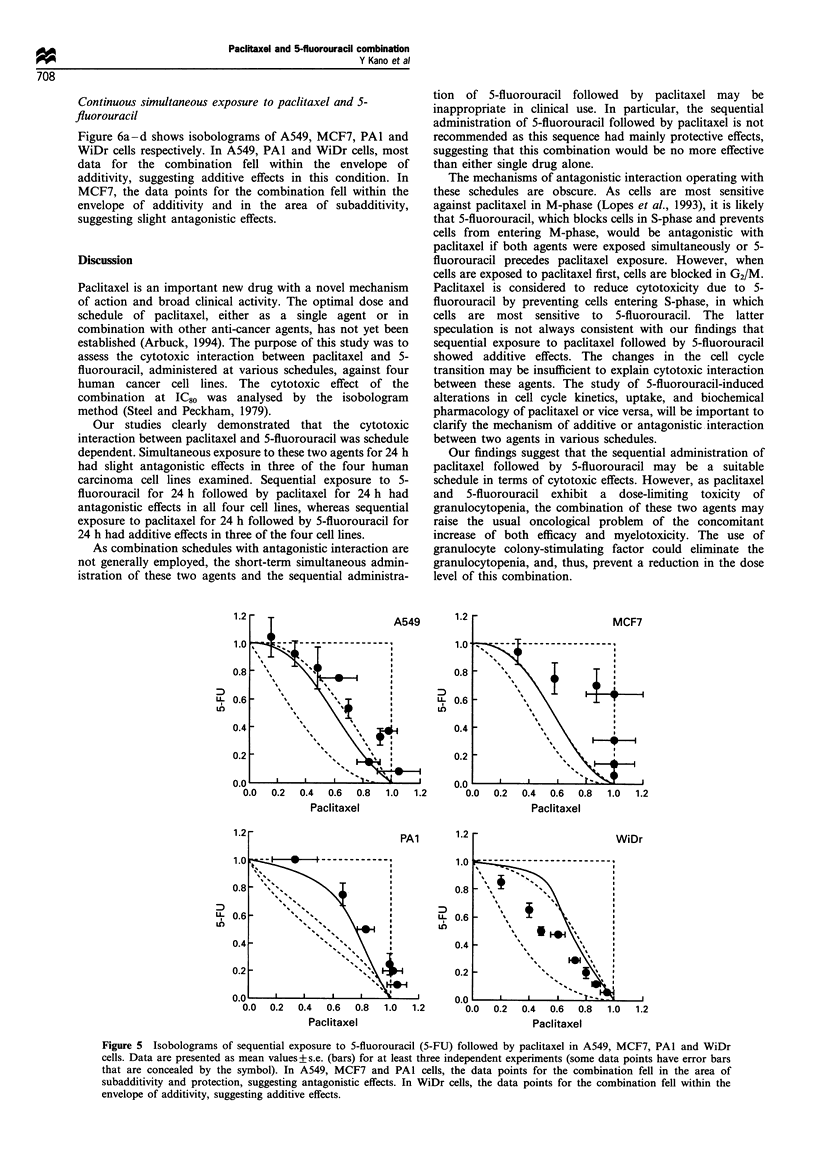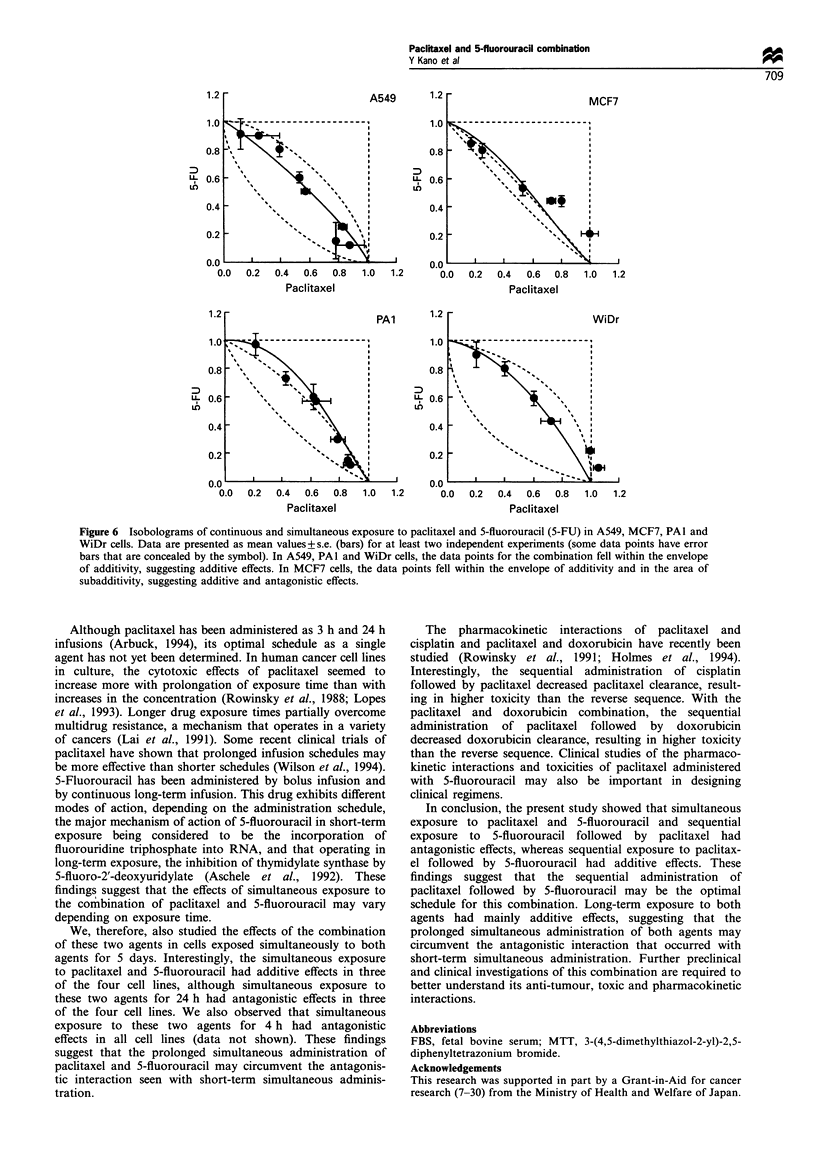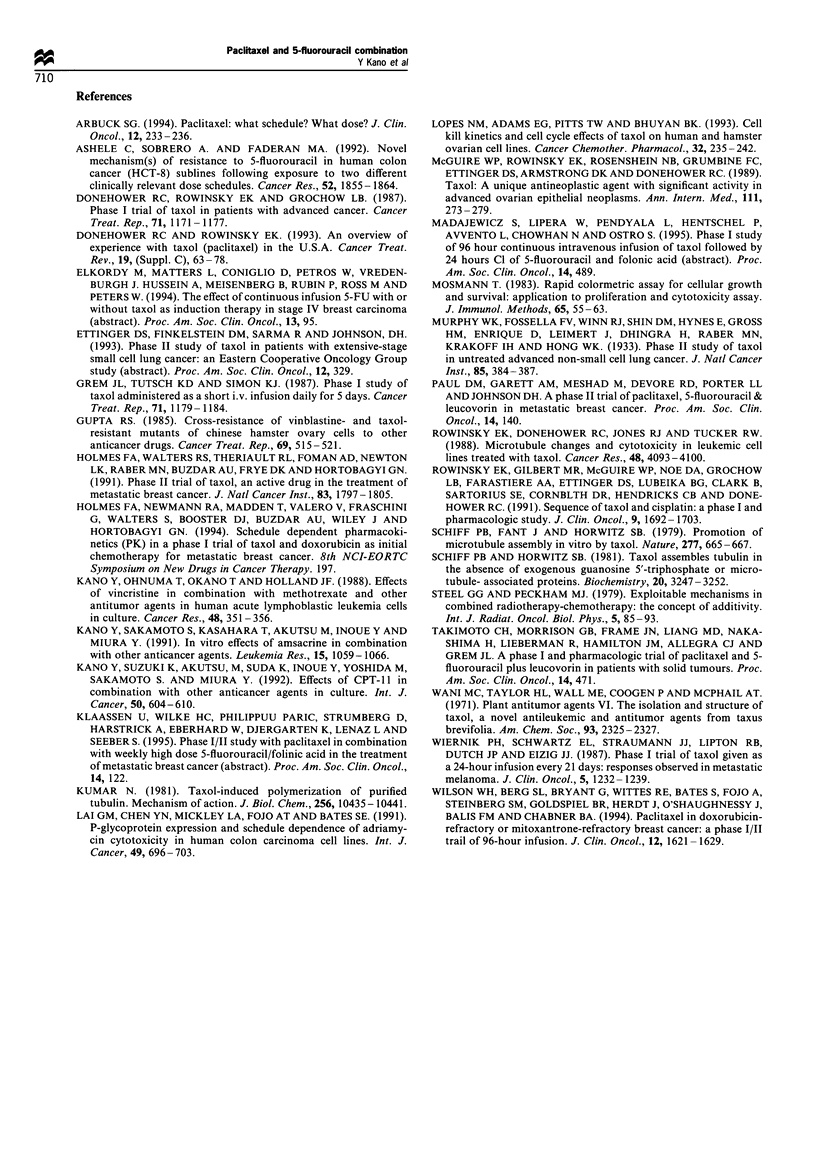# Schedule-dependent interaction between paclitaxel and 5-fluorouracil in human carcinoma cell lines in vitro.

**DOI:** 10.1038/bjc.1996.425

**Published:** 1996-09

**Authors:** Y. Kano, M. Akutsu, S. Tsunoda, J. Ando, J. Matsui, K. Suzuki, T. Ikeda, Y. Inoue, K. Adachi

**Affiliations:** Division of Medical Oncology, Tochigi Cancer Center, Japan.

## Abstract

We assessed the cytotoxic interaction between paclitaxel and 5-fluorouracil administered at various schedules against four human carcinoma cell lines, A549, MCF7, PA1 and WiDr. The cells were exposed simultaneously to paclitaxel and to 5-fluorouracil for 24 h or sequentially to one drug for 24 h followed by the other for 24 h, after which they were incubated in drug-free medium for 4 and 3 days respectively. In another experiment, the cells were exposed simultaneously to both agents for 5 days. Cell growth inhibition was determined by MTT reduction assay. The effects of drug combinations at IC80 were analysed by the isobologram. The cytotoxic interaction of paclitaxel and 5-fluorouracil was definitely schedule dependent. Simultaneous exposure to paclitaxel and 5-fluorouracil for 24 h showed mainly subadditive effects in A549, MCF7 and WiDr cell lines, whereas it showed additive effects in PA1 cells. Sequential exposure to paclitaxel followed by 5-fluorouracil showed additive effects in all cell lines. Sequential exposure to 5-fluorouracil followed by paclitaxel showed subadditive effects in A549, MCF7 and PA1 cells. Whereas it showed additive effects in WiDr cells. These findings suggest that maximum cytotoxic effects can be obtained when paclitaxel precedes 5-fluorouracil. Interestingly, the continuous (5-day) exposure to paclitaxel and 5-fluorouracil had additive effects in A549, PA1 and WiDr cells, indicating that the prolonged simultaneous administration of these agents may circumvent the antagonistic interaction produced by short-term simultaneous administration. These findings may be useful in clinical trials of combination chemotherapy with paclitaxel and 5-fluorouracil.


British Journal of Cancer (1996) 74, 704-710
© 1996 Stockton Press All rights reserved 0007-0920/96 $12.00

### Schedule-dependent interaction between paclitaxel and 5-fluorouracil in human carcinoma cell lines *in vitro*

Y Kano[1,3], M Akutsu[1], S Tsunoda[1], J Ando[2], J Matsui[2], K Suzuki[3], T Ikeda[4], Y Inoue[5] and KI Adachi[6]

*Divisions of [1]Medical Oncology, [2]Surgery and [3]Laboratory Medicine, Tochigi Cancer Center, Yonan 4-9-13, Utsunomiya, Tochigi, 320, Japan; Departments of [4]Surgery and [5]Plastic Surgery, School of Medicine, Keio University, Shinano-machi 35, Shinjuku-Ku, Tokyo, 160, Japan. [6]Medical Data Center, Pharmaceuticals Group, Nippon Kayaku Co., Ltd. 31-12, Shimo 3, Kita-ku, Tokyo, 115, Japan.*

**Summary** We assessed the cytotoxic interaction between paclitaxel and 5-fluorouracil administered at various schedules against four human carcinoma cell lines, A549, MCF7, PA1 and WiDr. The cells were exposed simultaneously to paclitaxel and to 5-fluorouracil for 24 h or sequentially to one drug for 24 h followed by the other for 24 h, after which they were incubated in drug-free medium for 4 and 3 days respectively. In another experiment, the cells were exposed simultaneously to both agents for 5 days. Cell growth inhibition was determined by MTT reduction assay. The effects of drug combinations at $IC_{80}$ were analysed by the isobologram. The cytotoxic interaction of paclitaxel and 5-fluorouracil was definitely schedule dependent. Simultaneous exposure to paclitaxel and 5-fluorouracil for 24 h showed mainly subadditive effects in A549, MCF7 and WiDr cell lines, whereas it showed additive effects in PA1 cells. Sequential exposure to paclitaxel followed by 5-fluorouracil showed additive effects in all cell lines. Sequential exposure to 5-fluorouracil followed by paclitaxel showed subadditive effects in A549, MCF7 and PA1 cells. Whereas it showed additive effects in WiDr cells. These findings suggest that maximum cytotoxic effects can be obtained when paclitaxel precedes 5-fluorouracil. Interestingly, the continuous (5-day) exposure to paclitaxel and 5-fluorouracil had additive effects in A549, PA1 and WiDr cells, indicating that the prolonged simultaneous administration of these agents may circumvent the antagonistic interaction produced by short-term simultaneous administration. These findings may be useful in clinical trials of combination chemotherapy with paclitaxel and 5-fluorouracil.

**Keywords:** paclitaxel; 5-fluorouracil; drug combination

Paclitaxel (taxol), a plant product isolated from the bark of the Pacific yew, *Taxus brevifolia* (Wani *et al.*, 1971), is a novel antimicrotubular agent that binds to the microtubules, promotes microtubule assembly and stabilises tubulin polymer formation (Schiff *et al.*, 1979, 1981; Kumar *et al.*, 1981). In clinical studies, the dose-limiting toxicity of paclitaxel was shown to be granulocytopenia; other toxic effects shown were hypersensitivity reaction, neuropathy, mucositis, slight nausea and vomiting, and slight cardiac injury (Grem *et al.*, 1987; Donehower *et al.*, 1987; Wiernilk *et al.*, 1987). Significant paclitaxel anti-tumour activity has been reported in patients with ovarian, breast, lung, head and neck, and oesophageal cancers (McGuire *et al.*, 1989; Holms *et al.*, 1991; Donehower *et al.*, 1993; Ettinger *et al.*, 1993; Murphy *et al.*, 1993). Its impressive clinical activity has prompted considerable interest in combining this drug with other anti-tumour agents. Clinical trials of its anti-tumour effects in combination with several other anti-tumour agents are now in progress.

For many years, 5-fluorouracil has been the mainstay of therapy for patients with solid tumours. 5-Fluorouracil, alone or in combination with other agents, is widely used in the treatment of gastrointestinal, breast and lung cancers–all non-curable diseases. It would be attractive to use paclitaxel and 5-fluorouracil in combination against these malignancies. As paclitaxel exerts its cytotoxic effects through a mechanism different from that of 5-fluorouracil, and it has no cross-resistance with 5-fluorouracil (Gupta, 1983), it appears reasonable to attempt to use these drugs in combination. Clinical trials of the combination of paclitaxel and 5-fluorouracil in a variety of schedules have, indeed, been initiated (Elkordy *et al.*, 1994; Klaassen *et al.*, 1994; Madajewicz *et al.*, 1995; Paul *et al.*, 1995; Takimoto *et al.*, 1995). However, it has not been determined whether paclitaxel and 5-fluorouracil should be given concomitantly or sequentially, or what the optimal infusion durations of each drug are. It is important to identify the optimal schedule for this combination; hence, in the present study, we attempted to elucidate the cytotoxic effects of paclitaxel and 5-fluorouracil combinations administered at various schedules in four human carcinoma cell lines.

Correspondence: Y Kano, Division of Medical Oncology, Tochigi Cancer Center, Yonan 4-9-13, Utsunomiya, Tochigi, 320, Japan
Received 12 June 1995; revised 20 December 1995; accepted 1 April 1996

### Materials and methods

### *Cell lines*

Experiments were conducted with four human carcinoma cell lines; non-small-cell lung cancer cells, A549, breast cancer cells, MCF7, ovarian cancer cells, PA1 and the colon cancer cells, WiDr. These cells were obtained from the American Type Culture Collection (Rockville, MD, USA) and maintained in 75 $cm^3$ plastic tissue culture flasks containing RPMI-1640 medium (Grand Island Biological Co., Grand Island, NY, USA) supplemented with 10% heat-inactivated fetal bovine serum (FBS) (Grand Island Biological Co.) and antibiotics. The doubling times of A549, MCF7, PA1 and WiDr cells in our experimental conditions were 30, 27, 24 and 27 h respectively.

### *Drugs*

Paclitaxel and 5-fluorouracil were provided by Bristol Myers Squibb Japan (Tokyo), and Kyowa Hakko (Tokyo) respectively. Paclitaxel was dissolved in dimethyl sulphoxide (Sigma, St Louis, MO, USA) and 5-fluorouracil was dissolved in RPMI-1640. The drugs were diluted with RPMI-1640 plus 10% FBS. The final concentration of dimethyl sulphoxide in the media was less than 0.05% and it had no effect on cell growth inhibition.

*Inhibition of cell growth by the combination of paclitaxel and 5-fluorouracil*

Exponentially growing cells were harvested with trypsin (0.05%)/EDTA (0.02%) and resuspended, to a final concentration of $5.0 \times 10^3$ cells $ml^{-1}$, in fresh medium containing 10% FBS and antibiotics. Aliquots of cell suspensions (100 $\mu$l) were dispensed with a multichannel pipette into the individual wells of a 96-well tissue culture plate with lid (Falcon, Oxnard, CA, USA). Each plate had one eight-well control column containing medium alone and one eight-well control column containing cells but no drug. Four plates were prepared for each drug combination schedule in each cell line. The cells were reincubated overnight in a humidified atmosphere containing 5% carbon dioxide at 37°C to allow for attachment.

*Simultaneous exposure (24 h) to paclitaxel and 5-fluorouracil* - After 20 to 24 h incubation, aliquots (50 $\mu$l) of each drug solution, at different concentrations, were added to individual wells (paclitaxel preceding 5-fluorouracil by approximately 10 min). The plates were then incubated under the same conditions for 24 h. After treatment, the cells were washed once with culture medium containing 1% FBS, and fresh medium (200 $\mu$l) was provided. The cells were then incubated again for 4 days.

*Sequential exposure to paclitaxel and 5-fluorouracil* After 20 to 24 h incubation, aliquots of media containing 10% FBS (50 $\mu$l) and solutions of paclitaxel (or 5-fluorouracil) (50 $\mu$l) at different concentrations were added to individual wells. The plates were then incubated under the same conditions for 24 h. The cells were washed once with culture medium containing 1% FBS and fresh medium containing 10% FBS (150 $\mu$l) and antibiotics, and aliquots of 5-fluorouracil (or paclitaxel) solution (50 $\mu$l) at different concentrations were added. The plates were incubated again under the same conditions for 24 h. After treatment, the cells were washed once, and fresh medium (200 $\mu$l) was provided. The cells were then incubated again for 3 days.

*Continuous and simultaneous exposure to paclitaxel and 5-fluorouracil* After 20 to 24 h incubation, aliquots of paclitaxel (50 $\mu$l) and 5-fluorouracil (50 $\mu$l) solution at different concentrations were added to individual wells. The plates were then incubated under the same conditions for 5 days.

*MTT assay*

Viable cell growth was determined by MTT reduction assay (Mosmann, 1983) as described previously (Kano *et al.*, 1991). Aliquots of 50 $\mu$l of MTT (1 mg $ml^{-1}$) were added to each well. After 4 h at 37°C, the supernatant was removed. Dimethyl sulphoxide (150 $\mu$l) was then added and the plates were vigorously shaken to solubilise the MTT–formazan product. Absorbance at 570 nm was measured with a Titertek multiscan. For all cell lines studied, we established a linear relation between the MTT assay and cell number within the range of the experiments shown.

*Data analysis*

Dose–response curves were plotted on a semilog scale as a percentage of the control, the cell number of which was obtained from samples with no drug exposure that were processed simultaneously. Dose–response interactions between paclitaxel and 5-fluorouracil at the point of $IC_{80}$ were evaluated by the isobologram (Steel and Peckham, 1979). The $IC_{80}$ was defined as the concentration of drug that produced 80% cell growth inhibition; 80% reduction of absorbance. We used $IC_{80}$ instead of the more common $IC_{50}$ as the combined effects at $IC_{50}$ were sometimes different from those of $IC_{80}$, which would be more important than $IC_{50}$ in this study.

Figure 1 shows a schematic isobologram. The theoretical basis of the isobologram has been described previously (Steel and Peckham, 1979; Kano *et al.*, 1988, 1992). Three isoeffect curves were constructed based upon the dose–response curves of paclitaxel and 5-fluorouracil.

(1) Mode I line (solid line in Figure 1): When the dose of paclitaxel was chosen, an incremental effect remained to be produced by 5-fluorouracil. The addition was calculated by taking the increment in doses, starting from zero, that produced log survivals that added up to $IC_{80}$ (heteroaddition). If the agents are acting additively by independent mechanism, combined data points would lie near the mode I line.

(2) Mode II (a) line (dotted line in Figure 1). When the dose of paclitaxel was chosen, an incremental effect remained to be produced by 5-fluorouracil. The addition was calculated by taking the increment in doses, starting from the point on the dose–response curve of paclitaxel where the effect of paclitaxel had ended, that produced log survivals that added up to $IC_{80}$ (isoaddition).

(3) Mode II (b) line (dotted line in Figure 1). Similarly to procedure for the mode II (a) line, when the dose of 5-fluorouracil was chosen, an incremental effect remained to be produced by paclitaxel. The addition was calculated by taking the increment in doses, starting from the point on the dose–response curve of 5-fluorouracil where its effect had ended, that produced log survivals that added up to $IC_{80}$ (isoaddition). If the agents are acting additively by similar mechanism, combined data points would lie near mode II lines.

As we cannot know whether the combined effects of two agents will be heteroadditive, isoadditive or intermediate between these extremes, all possibilities should be considered. Thus, when the data points of the drug combination fell within the area surrounded by three lines (envelope of additivity) (Pb in Figure 1), the combination was regarded as additive. We used this envelope not only to evaluate simultaneous exposure to the paclitaxel and 5-fluorouracil combination, but also to evaluate the sequential exposure to both agents as the cytotoxicity of the first agent could be modulated by the second agent under our experimental conditions.

In this isobologram, an additive effect indicates great superiority of the combination to a single agent, even though the data points for the combination do not reach

**Figure 1** Schematic representation of isobologram. Envelope of additivity [hatched symbol], surrounded by mode I (solid line) and mode II (dotted lines) isobologram lines, was constructed from the dose–response curves of paclitaxel and 5-fluorouracil (5-FU). The concentrations that produced 80% cell growth inhibition were expressed as 1.0 in the ordinate and the abscissa of isobolograms. Combined data points Pa, Pb, Pc, and Pd show supra-additive, additive, subadditive and protective effects respectively.

**Figure 2** Dose–response curves for paclitaxel alone, 5-fluorouracil (5-FU) alone, and their combinations in MCF7 cells. (**a**) Simultaneous exposure (24 h) to paclitaxel and 5-fluorouracil. (**b**) Sequential exposure to paclitaxel (24 h) followed by 5-fluorouracil (24 h). (**c**) Sequential exposure to 5-fluorouracil (24 h) followed by paclitaxel (24 h). Cell numbers were measured by MTT assay after 5 days and were plotted as a percentage of the control (cells not exposed to drugs). Paclitaxel concentrations are shown on the abscissa. 5-Fluorouracil concentrations for each symbol are in the upper right of panel (**a**). Each point represents the mean value for at least three independent experiments performed in quadruplicate; the s.e. of the means were less than 11% and were then omitted.

the supra-additive area. Thus, anti-cancer agents that show additive effects should have greater cytotoxic effects in combinations than when used as single agents. When the data points fell to the left of the envelope (i.e. the effect of the combination was caused by lower doses of the two agents than predicted) (Pa in Figure 1), we regarded the drugs as having a supra-additive effect (synergism). When the points fell to the right of the envelope (i.e. the effect of the combination was caused by higher doses of the two agents than predicted), but within the square or on the line of the square (Pc in Figure 1), we regarded the two drugs as having a subadditive effect, that is, the combination was superior or equal to a single agent but was less than additive. When the data points were outside the square (Pd in Figure 1), the combination was regarded as having a protective effect, i.e. the combination was inferior in cytotoxic action to a single agent. Both subadditive and protective interaction were regarded as antagonism.

### Results

Figure 2a–c shows dose–response curves for MCF7 cells exposed to paclitaxel and 5-fluorouracil for 24 h at various schedules; simultaneous exposure to drugs, sequential exposure to paclitaxel followed by 5-fluorouracil and sequential exposure to 5-fluorouracil followed by paclitaxel respectively. Paclitaxel concentrations are shown on the abscissa. Dose–response curves, in which 5-fluorouracil concentrations are shown on the abscissa, can be made based on the same data (figure not shown). Isobolograms at the $IC_{80}$ level were generated using these dose–response curves for the combinations.

### *Simultaneous exposure (24 h) to paclitaxel and 5-fluorouracil*

Figure 3a–d shows isobolograms of A549, MCF7, PA1 and WiDr cells respectively, simultaneously exposed to paclitaxel and 5-fluorouracil. In A549, MCF7 and WiDr cells, the data points for the combination fell mainly in the area of subadditivity; these findings we regarded as indicative of simultaneous exposure produced slight antagonistic effects. In PA1 cells, the data points for the combination fell within the envelope of additivity, suggesting that the combination had additive effects.

### *Sequential exposure to paclitaxel followed by 5-fluorouracil*

Figure 4a–d shows isobolograms of A549, MCF7, PA1 and WiDr cells respectively. In this experimental condition, all cell lines showed similar effects; most of the data points for the combination fell within the envelope of additivity, suggesting that sequential exposure to paclitaxel followed by 5-fluorouracil produced additive effects.

### *Sequential exposure to 5-fluorouracil followed by paclitaxel*

Figure 5a–d shows isobolograms of A549, MCF7, PA1 and WiDr cells respectively. In A549, MCF7 and PA1 cells, the data points for the combination fell mainly in the area of subadditivity and protection, suggesting antagonistic effects in this condition. In WiDr cells, the data points for the combination fell within the envelope of additivity, suggesting additive effects.

**Figure 3** Isobolograms of simultaneous exposure to paclitaxel and 5-fluorouracil (5-FU) in A549, MCF7, PA1 and WiDr cells. Data are presented as mean values±s.e. (bars) for at least three independent experiments (some data points have error bars that are concealed by the symbol). In A549, MCF7 and WiDr cells, the data points of the combinations fell mainly in the area of subadditivity, suggesting that the combination showed slight antagonistic effects. In PA-1 cells, the data points fell within the envelope of additivity, suggesting additive effects.

**Figure 4** Isobolograms of sequential exposure to paclitaxel followed by 5-fluorouracil (5-FU) in A549, MCF7, PA1 and WiDr cells. Data are presented as mean values±s.e. (bars) for at least three independent experiments (some data points have error bars that are concealed by the symbol). The data points for the combination fell within the envelope of additivity for all four cell lines, suggesting additive effects.

*Continuous simultaneous exposure to paclitaxel and 5-fluorouracil*

Figure 6a–d shows isobolograms of A549, MCF7, PA1 and WiDr cells respectively. In A549, PA1 and WiDr cells, most data for the combination fell within the envelope of additivity, suggesting additive effects in this condition. In MCF7, the data points for the combination fell within the envelope of additivity and in the area of subadditivity, suggesting slight antagonistic effects.

### Discussion

Paclitaxel is an important new drug with a novel mechanism of action and broad clinical activity. The optimal dose and schedule of paclitaxel, either as a single agent or in combination with other anti-cancer agents, has not yet been established (Arbuck, 1994). The purpose of this study was to assess the cytotoxic interaction between paclitaxel and 5-fluorouracil, administered at various schedules, against four human cancer cell lines. The cytotoxic effect of the combination at $IC_{80}$ was analysed by the isobologram method (Steel and Peckham, 1979).

Our studies clearly demonstrated that the cytotoxic interaction between paclitaxel and 5-fluorouracil was schedule dependent. Simultaneous exposure to these two agents for 24 h had slight antagonistic effects in three of the four human carcinoma cell lines examined. Sequential exposure to 5-fluorouracil for 24 h followed by paclitaxel for 24 h had antagonistic effects in all four cell lines, whereas sequential exposure to paclitaxel for 24 h followed by 5-fluorouracil for 24 h had additive effects in three of the four cell lines.

As combination schedules with antagonistic interaction are not generally employed, the short-term simultaneous administration of these two agents and the sequential administration of 5-fluorouracil followed by paclitaxel may be inappropriate in clinical use. In particular, the sequential administration of 5-fluorouracil followed by paclitaxel is not recommended as this sequence had mainly protective effects, suggesting that this combination would be no more effective than either single drug alone.

The mechanisms of antagonistic interaction operating with these schedules are obscure. As cells are most sensitive against paclitaxel in M-phase (Lopes *et al.*, 1993), it is likely that 5-fluorouracil, which blocks cells in S-phase and prevents cells from entering M-phase, would be antagonistic with paclitaxel if both agents were exposed simultaneously or 5-fluorouracil precedes paclitaxel exposure. However, when cells are exposed to paclitaxel first, cells are blocked in $G_2$/M. Paclitaxel is considered to reduce cytotoxicity due to 5-fluorouracil by preventing cells entering S-phase, in which cells are most sensitive to 5-fluorouracil. The latter speculation is not always consistent with our findings that sequential exposure to paclitaxel followed by 5-fluorouracil showed additive effects. The changes in the cell cycle transition may be insufficient to explain cytotoxic interaction between these agents. The study of 5-fluorouracil-induced alterations in cell cycle kinetics, uptake, and biochemical pharmacology of paclitaxel or vice versa, will be important to clarify the mechanism of additive or antagonistic interaction between two agents in various schedules.

Our findings suggest that the sequential administration of paclitaxel followed by 5-fluorouracil may be a suitable schedule in terms of cytotoxic effects. However, as paclitaxel and 5-fluorouracil exhibit a dose-limiting toxicity of granulocytopenia, the combination of these two agents may raise the usual oncological problem of the concomitant increase of both efficacy and myelotoxicity. The use of granulocyte colony-stimulating factor could eliminate the granulocytopenia, and, thus, prevent a reduction in the dose level of this combination.

**Figure 5** Isobolograms of sequential exposure to 5-fluorouracil (5-FU) followed by paclitaxel in A549, MCF7, PA1 and WiDr cells. Data are presented as mean values ± s.e. (bars) for at least three independent experiments (some data points have error bars that are concealed by the symbol). In A549, MCF7 and PA1 cells, the data points for the combination fell in the area of subadditivity and protection, suggesting antagonistic effects. In WiDr cells, the data points for the combination fell within the envelope of additivity, suggesting additive effects.

**Figure 6** Isobolograms of continuous and simultaneous exposure to paclitaxel and 5-fluorouracil (5-FU) in A549, MCF7, PA1 and WiDr cells. Data are presented as mean values ± s.e. (bars) for at least two independent experiments (some data points have error bars that are concealed by the symbol). In A549, PA1 and WiDr cells, the data points for the combination fell within the envelope of additivity, suggesting additive effects. In MCF7 cells, the data points fell within the envelope of additivity and in the area of subadditivity, suggesting additive and antagonistic effects.

Although paclitaxel has been administered as 3 h and 24 h infusions (Arbuck, 1994), its optimal schedule as a single agent has not yet been determined. In human cancer cell lines in culture, the cytotoxic effects of paclitaxel seemed to increase more with prolongation of exposure time than with increases in the concentration (Rowinsky *et al.*, 1988; Lopes *et al.*, 1993). Longer drug exposure times partially overcome multidrug resistance, a mechanism that operates in a variety of cancers (Lai *et al.*, 1991). Some recent clinical trials of paclitaxel have shown that prolonged infusion schedules may be more effective than shorter schedules (Wilson *et al.*, 1994). 5-Fluorouracil has been administered by bolus infusion and by continuous long-term infusion. This drug exhibits different modes of action, depending on the administration schedule, the major mechanism of action of 5-fluorouracil in short-term exposure being considered to be the incorporation of fluorouridine triphosphate into RNA, and that operating in long-term exposure, the inhibition of thymidylate synthase by 5-fluoro-2′-deoxyuridylate (Aschele *et al.*, 1992). These findings suggest that the effects of simultaneous exposure to the combination of paclitaxel and 5-fluorouracil may vary depending on exposure time.

We, therefore, also studied the effects of the combination of these two agents in cells exposed simultaneously to both agents for 5 days. Interestingly, the simultaneous exposure to paclitaxel and 5-fluorouracil had additive effects in three of the four cell lines, although simultaneous exposure to these two agents for 24 h had antagonistic effects in three of the four cell lines. We also observed that simultaneous exposure to these two agents for 4 h had antagonistic effects in all cell lines (data not shown). These findings suggest that the prolonged simultaneous administration of paclitaxel and 5-fluorouracil may circumvent the antagonistic interaction seen with short-term simultaneous administration.

The pharmacokinetic interactions of paclitaxel and cisplatin and paclitaxel and doxorubicin have recently been studied (Rowinsky *et al.*, 1991; Holmes *et al.*, 1994). Interestingly, the sequential administration of cisplatin followed by paclitaxel decreased paclitaxel clearance, resulting in higher toxicity than the reverse sequence. With the paclitaxel and doxorubicin combination, the sequential administration of paclitaxel followed by doxorubicin decreased doxorubicin clearance, resulting in higher toxicity than the reverse sequence. Clinical studies of the pharmacokinetic interactions and toxicities of paclitaxel administered with 5-fluorouracil may also be important in designing clinical regimens.

In conclusion, the present study showed that simultaneous exposure to paclitaxel and 5-fluorouracil and sequential exposure to 5-fluorouracil followed by paclitaxel had antagonistic effects, whereas sequential exposure to paclitaxel followed by 5-fluorouracil had additive effects. These findings suggest that the sequential administration of paclitaxel followed by 5-fluorouracil may be the optimal schedule for this combination. Long-term exposure to both agents had mainly additive effects, suggesting that the prolonged simultaneous administration of both agents may circumvent the antagonistic interaction that occurred with short-term simultaneous administration. Further preclinical and clinical investigations of this combination are required to better understand its anti-tumour, toxic and pharmacokinetic interactions.

**Abbreviations**

FBS, fetal bovine serum; MTT, 3-(4,5-dimethylthiazol-2-yl)-2,5-diphenyltetrazonium bromide.

**Acknowledgements**

This research was supported in part by a Grant-in-Aid for cancer research (7–30) from the Ministry of Health and Welfare of Japan.

### References

ARBUCK SG. (1994). Paclitaxel: what schedule? What dose? *J. Clin. Oncol.*, **12**, 233–236.

ASHELE C, SOBRERO A. AND FADERAN MA. (1992). Novel mechanism(s) of resistance to 5-fluorouracil in human colon cancer (HCT-8) sublines following exposure to two different clinically relevant dose schedules. *Cancer Res.*, **52**, 1855–1864.

DONEHOWER RC, ROWINSKY EK AND GROCHOW LB. (1987). Phase I trial of taxol in patients with advanced cancer. *Cancer Treat. Rep.*, **71**, 1171–1177.

DONEHOWER RC AND ROWINSKY EK. (1993). An overview of experience with taxol (paclitaxel) in the U.S.A. *Cancer Treat. Rev.*, **19**, (Suppl. C), 63–78.

ELKORDY M, MATTERS L, CONIGLIO D, PETROS W, VREDENBURGH J. HUSSEIN A, MEISENBERG B, RUBIN P, ROSS M AND PETERS W. (1994). The effect of continuous infusion 5-FU with or without taxol as induction therapy in stage IV breast carcinoma (abstract). *Proc. Am. Soc. Clin. Oncol.*, **13**, 95.

ETTINGER DS, FINKELSTEIN DM, SARMA R AND JOHNSON, DH. (1993). Phase II study of taxol in patients with extensive-stage small cell lung cancer: an Eastern Cooperative Oncology Group study (abstract). *Proc. Am. Soc. Clin. Oncol.*, **12**, 329.

GREM JL, TUTSCH KD AND SIMON KJ. (1987). Phase I study of taxol administered as a short i.v. infusion daily for 5 days. *Cancer Treat. Rep.*, **71**, 1179–1184.

GUPTA RS. (1985). Cross-resistance of vinblastine- and taxol-resistant mutants of chinese hamster ovary cells to other anticancer drugs. *Cancer Treat. Rep.*, **69**, 515–521.

HOLMES FA, WALTERS RS, THERIAULT RL, FOMAN AD, NEWTON LK, RABER MN, BUZDAR AU, FRYE DK AND HORTOBAGYI GN. (1991). Phase II trial of taxol, an active drug in the treatment of metastatic breast cancer. *J. Natl Cancer Inst.*, **83**, 1797–1805.

HOLMES FA, NEWMANN RA, MADDEN T, VALERO V, FRASCHINI G, WALTERS S, BOOSTER DJ, BUZDAR AU, WILEY J AND HORTOBAGYI GN. (1994). Schedule dependent pharmacokinetics (PK) in a phase I trial of taxol and doxorubicin as initial chemotherapy for metastatic breast cancer. *8th NCI-EORTC Symposium on New Drugs in Cancer Therapy.* 197.

KANO Y, OHNUMA T, OKANO T AND HOLLAND JF. (1988). Effects of vincristine in combination with methotrexate and other antitumor agents in human acute lymphoblastic leukemia cells in culture. *Cancer Res.*, **48**, 351–356.

KANO Y, SAKAMOTO S, KASAHARA T, AKUTSU M, INOUE Y AND MIURA Y. (1991). In vitro effects of amsacrine in combination with other anticancer agents. *Leukemia Res.*, **15**, 1059–1066.

KANO Y, SUZUKI K, AKUTSU, M, SUDA K, INOUE Y, YOSHIDA M, SAKAMOTO S. AND MIURA Y. (1992). Effects of CPT-11 in combination with other anticancer agents in culture. *Int. J. Cancer*, **50**, 604–610.

KLAASSEN U, WILKE HC, PHILIPPUU PARIC, STRUMBERG D, HARSTRICK A, EBERHARD W, DJERGARTEN K, LENAZ L AND SEEBER S. (1995). Phase I/II study with paclitaxel in combination with weekly high dose 5-fluorouracil/folinic acid in the treatment of metastatic breast cancer (abstract). *Proc. Am. Soc. Clin. Oncol.*, **14**, 122.

KUMAR N. (1981). Taxol-induced polymerization of purified tubulin. Mechanism of action. *J. Biol. Chem.*, **256**, 10435–10441.

LAI GM, CHEN YN, MICKLEY LA, FOJO AT AND BATES SE. (1991). P-glycoprotein expression and schedule dependence of adriamycin cytotoxicity in human colon carcinoma cell lines. *Int. J. Cancer*, **49**, 696–703.

LOPES NM, ADAMS EG, PITTS TW AND BHUYAN BK. (1993). Cell kill kinetics and cell cycle effects of taxol on human and hamster ovarian cell lines. *Cancer Chemother. Pharmacol.*, **32**, 235–242.

McGUIRE WP, ROWINSKY EK, ROSENSHEIN NB, GRUMBINE FC, ETTINGER DS, ARMSTRONG DK AND DONEHOWER RC. (1989). Taxol: A unique antineoplastic agent with significant activity in advanced ovarian epithelial neoplasms. *Ann. Intern. Med.*, **111**, 273–279.

MADAJEWICZ S, LIPERA W, PENDYALA L, HENTSCHEL P, AVVENTO L, CHOWHAN N AND OSTRO S. (1995). Phase I study of 96 hour continuous intravenous infusion of taxol followed by 24 hours Cl of 5-fluorouracil and folonic acid (abstract). *Proc. Am. Soc. Clin. Oncol.*, **14**, 489.

MOSMANN T. (1983). Rapid colormetric assay for cellular growth and survival: application to proliferation and cytotoxicity assay. *J. Immunol. Methods*, **65**, 55–63.

MURPHY WK, FOSSELLA FV, WINN RJ, SHIN DM, HYNES E, GROSS HM, ENRIQUE D, LEIMERT J, DHINGRA H, RABER MN, KRAKOFF IH AND HONG WK. (1933). Phase II study of taxol in untreated advanced non-small cell lung cancer. *J. Natl Cancer Inst.*, **85**, 384–387.

PAUL DM, GARETT AM, MESHAD M, DEVORE RD, PORTER LL AND JOHNSON DH. A phase II trial of paclitaxel, 5-fluorouracil & leucovorin in metastatic breast cancer. *Proc. Am. Soc. Clin. Oncol.*, **14**, 140.

ROWINSKY EK, DONEHOWER RC, JONES RJ AND TUCKER RW. (1988). Microtubule changes and cytotoxicity in leukemic cell lines treated with taxol. *Cancer Res.*, **48**, 4093–4100.

ROWINSKY EK, GILBERT MR, McGUIRE WP, NOE DA, GROCHOW LB, FARASTIERE AA, ETTINGER DS, LUBEIKA BG, CLARK B, SARTORIUS SE, CORNBLTH DR, HENDRICKS CB AND DONEHOWER RC. (1991). Sequence of taxol and cisplatin: a phase I and pharmacologic study. *J. Clin. Oncol.*, **9**, 1692–1703.

SCHIFF PB, FANT J AND HORWITZ SB. (1979). Promotion of microtubule assembly in vitro by taxol. *Nature*, **277**, 665–667.

SCHIFF PB AND HORWITZ SB. (1981). Taxol assembles tubulin in the absence of exogenous guanosine 5′-triphosphate or microtubule- associated proteins. *Biochemistry*, **20**, 3247–3252.

STEEL GG AND PECKHAM MJ. (1979). Exploitable mechanisms in combined radiotherapy-chemotherapy: the concept of additivity. *Int. J. Radiat. Oncol. Biol. Phys.*, **5**, 85–93.

TAKIMOTO CH, MORRISON GB, FRAME JN, LIANG MD, NAKASHIMA H, LIEBERMAN R, HAMILTON JM, ALLEGRA CJ AND GREM JL. A phase I and pharmacologic trial of paclitaxel and 5-fluorouracil plus leucovorin in patients with solid tumours. *Proc. Am. Soc. Clin. Oncol.*, **14**, 471.

WANI MC, TAYLOR HL, WALL ME, COOGEN P AND MCPHAIL AT. (1971). Plant antitumor agents VI. The isolation and structure of taxol, a novel antileukemic and antitumor agents from taxus brevifolia. *Am. Chem. Soc.*, **93**, 2325–2327.

WIERNIK PH, SCHWARTZ EL, STRAUMANN JJ, LIPTON RB, DUTCH JP AND EIZIG JJ. (1987). Phase I trial of taxol given as a 24-hour infusion every 21 days: responses observed in metastatic melanoma. *J. Clin. Oncol.*, **5**, 1232–1239.

WILSON WH, BERG SL, BRYANT G, WITTES RE, BATES S, FOJO A, STEINBERG SM, GOLDSPIEL BR, HERDT J, O'SHAUGHNESSY J, BALIS FM AND CHABNER BA. (1994). Paclitaxel in doxorubicin-refractory or mitoxantrone-refractory breast cancer: a phase I/II trail of 96-hour infusion. *J. Clin. Oncol.*, **12**, 1621–1629.